# PU14, a Novel Matrix Protein, Participates in Pearl Oyster, *Pinctada Fucata*, Shell Formation

**DOI:** 10.1007/s10126-020-10014-3

**Published:** 2021-03-10

**Authors:** Yinghui Ji, Xue Yang, Dong Yang, Rongqing Zhang

**Affiliations:** 1 Ministry of Education Key Laboratory of Protein Sciences, School of Life Sciences, Tsinghua University, Beijing, 100084 China; 2 Zhe Jiang Provincial Key Laboratory of Applied Enzymology, Yangtze Delta Region Institute of Tsinghua University, 705 Yatai Road, Jiaxing, 314006 China; 3 College of Biological, Chemical Sciences and Engineering, Jiaxing University, Jiaxing, 314001 China

**Keywords:** Biomineralization, Shell matrix protein, *Pinctada fucata*, Calcite crystallization

## Abstract

Biomineralization is a widespread biological process, involved in the formation of shells, teeth, and bones. Shell matrix proteins have been widely studied for their importance during shell formation. In 2015, our group identified 72 unique shell matrix proteins in *Pinctada fucata*, among which PU14 is a matrix protein detected in the soluble fraction that solely exists in the prismatic layer. However, the function of PU14 is still unclear. In this study, the full-length cDNA sequence of PU14 was obtained and functional analyses of PU14 protein during shell formation were performed. The deduced protein has a molecular mass of 77.8 kDa and an isoelectric point of 11.34. The primary protein structure contains Gln-rich and random repeat units, which are typical characteristics of matrix protein and indicate its potential function during shell formation. In vivo and in vitro experiments indicated PU14 has prismatic layer functions during shell formation. The tissue expression patterns showed that *PU14* was mainly expressed in the mantle tissue, which is consistent with prismatic layer formation. Notching experiments suggested that *PU14* responded to repair and regenerate the injured shell. After inhibiting gene expression by injecting PU14-specific double-stranded RNA, the inner surface of the prismatic layer changed significantly and became rougher. Further, in vitro experiments showed that recombinant protein rPU14 impacted calcite crystal morphology. Taken together, characterization and functional analyses of a novel matrix protein, PU14, provide new insights about basic matrix proteins and their functions during shell formation.

## Introduction

Biomineralization is a widespread and an elaborately controlled process by which living organisms form various minerals (Boskey 1998). Biological structures, such as teeth, bone, spicules, shell, and pearl, are common biomineralization products and play multiple roles in feeding, defense, support, and locomotion (Li and Ortiz 2014; Kim et al. 2012; Singh et al. 2018).

Pearl oyster, *Pinctada fucata*, one of the most important cultured pearl species in China, is a well-studied model species for biomineralization investigations (Addadi et al. 2006; Liu et al. 2015a, b). Structurally, the shell of the pearl oyster includes two layers: prismatic and nacreous layers (Sudo et al. 1997). Each layer is made up of 95% calcium carbonate (CaCO_3_) and 5% organic macromolecules (Falini et al. 2013). As the major component of shell, calcium carbonate (CaCO_3_) mainly exists as calcite, aragonite, vaterite, or amorphous calcium carbonate (ACC), which is usually considered the biomineralization precursor (Falini, Fermani, and Ripamonti 2002; Goffredo et al. 2011; Marie et al. 2012).

Although organic macromolecules comprise only a small fraction of the weight, they play a vital role in the process (Miyazaki et al. 2010). Organic macromolecules include proteins, lipids (Farre and Dauphin 2009), and polysaccharides, but matrix proteins are thought to be the most important component in biomineralization regulation, affecting crystal nucleation, polymorphism (Takeuchi et al. 2008), orientation, and morphology (Alivisatos 2000; Qi et al. 2019; Feng et al. 2016; Chang et al. 2016). Previous studies have identified several matrix proteins that possess biomineralization functions (Fang et al. 2011; Su et al. 2013). For instance, Pif (Miyashita et al. 2000; Suzuki et al. 2009), Prismin (Takagi and Miyashita 2010; Perovic et al. 2013), Shematrin (Yano et al. 2006), and KRMP (Zhang et al. 2006; Liang et al. 2015, 2016) matrix protein families appear to play critical roles in regulating prismatic layer growth and building the shell’s insoluble organic frame. Prismalin-14(Gao et al. 2016) inhibits calcium carbonate precipitation in vitro. Protein pfN23(Fang et al. 2012) accelerates calcium carbonate deposition and leads to the formation of aragonite crystals. Further, other proteins perform negative functions. SPARC (Miyamoto et al. 2013), PfY2 (Yan et al. 2017) protein, and N25 (Yan et al. 2019) protein have been reported to stabilize vaterite, preventing it from forming other polymorphs. As reported, shell matrix proteins have some common characteristics. First, they usually possess signal peptides because they are secreted by the mantle tissue before functioning in shell formation. Second, their primary structures usually contain tandem-arranged repeat units.

Over the past 30 years, several traditional approaches have been used to understand mechanisms of shell formation (Checa et al. 2009; Kono et al. 2000). In the beginning, biochemical extractions were used to isolate proteins (Miyamoto et al. 1996), which were intensively studied. However, only matrix proteins with high expression level could be discovered this way. Then, researchers began to employ rapid amplification of cDNA end (RACE) techniques to screen for new matrix protein members on the basis of available protein sequences. For example, lysine (K)-rich matrix protein (KRMP) was identified from similarity with a protein sequence which included a high proportion of lysine, glycine, and tyrosine in this manner (Liang et al. 2015).

However, this approach is limited because it can only discover proteins that are similar to previously studied ones. Thus, new methods have been deployed over the past few years, including genomics, transcriptomics, and proteomics. In combination with a draft genome, Liu et al. identified 72 unique SMPs by liquid chromatography-tandem mass spectrometry (LC-MS/MS) analysis of proteins extracted from *P. fucata* shells (Liu et al. 2015a, b). Among the 72 identified matrix proteins, PU14 is one of the proteins that is only found in the prismatic layer and is not soluble in EDTA. Its primary sequence is characterized by Q-rich and tandem-arranged repeat units, which is an important feature of matrix protein.

In this study, we cloned a new matrix protein candidate gene, *PU14*, and identified the effects of its corresponding protein on CaCO_3_ crystallization during shell formation, particularly in the formation of prismatic layer. Functional assays, including expression pattern analysis and in vivo RNA interference (RNAi) assays, indicated PU14 is a matrix protein involved in shell formation, mainly in the prismatic layer. In vitro assays showed that purified recombinant rPU14 can affect CaCO_3_ crystallization, especially calcite crystal formation. These results provide new evidence on how matrix proteins regulate crystal growth and additional insight into shell formation.

## Material and Methods

### Methods Ethics Statement.

The study was approved by the Animal Ethics Committee of Tsinghua University, Beijing, China.

### Experimental Material.

Adult pearl oysters, *P. fucata*, with shell lengths around 6 cm and wet weight of 40 g, were from the Zhanjiang Pearl Farm (Guangdong, China). Oysters were cultivated in artificial seawater (Sude Instant Sea Salt, 3%) under a controlled temperature (20 ± 2 °C) before experiments. They were fed with yeast or spirulina powder dissolved in seawater every 3 days. Healthy individuals were selected randomly for experiments.

### Tissue Collection and Preparation.

Seven different tissues (mantle edge, mantle pallium, adductor muscle, gill, foot, viscus, and gonad) were removed from the oysters. The tissues were immediately flash-frozen in liquid nitrogen and were then powdered in liquid nitrogen for further experiments.

### RNA Extraction and cDNA Synthesis.

Total RNA of the seven tissues from different individuals (5 individuals in every group in RNAi experiments and 3 individuals in shell notching experiments) were extracted using TRIzol reagent (Invitrogen, USA) following the manufacturer’s instructions. A NanoDrop Lite spectrophotometer (Life Technologies, Thermo) was used to determine the quality and quantity of RNA by measuring the optical density at 260 nm and 280 nm.

### Isolation and Identification of Gene *PU14*

#### Cloning and Bioinformatic Analyses of the Complete PU14 cDNA Sequence

A cDNA template was synthesized from 500 ng of mantle RNA with a SMARTer rapid amplification of cDNA ends (RACE) Amplification Kit (Clontech, Japan). Then the SMARTer RACE cDNA Amplification kit (Clontech) was used to determine the full-length sequence of the *PU14* gene. Primers PU14-5R-1, PU14-5R-2, and PU14-5R-3 combined with the primers UPMlong, UPMshort, and NUP were used with primers supplied in the 5′RACE kit. Primers PU14-3R-1, PU14-3R-2, and PU14-3R-3 were used with primers supplied with the 3′RACE kit. Refer to Table 1 for primer details.

**Table 1:** Primer details. F, forward; R, reverse; RT, real-time PCR

Primers for RACE	
PU14-3R-1	CCTCAGTGGGGCAGGCTGTAATGCAGAC
PU14-3R-2	GGAGGTCAAGTTCCGACAATGGGCAAAG
PU14-3R-3	CAGGTAACCCCTGCCGGCCAAGTTGG
PU14-5R-1	CCATTGTTGCTGTTGAACCCACTGG
PU14-5R-2	GGCTGTACCATTTGAGATTGCTGGTTC
PU14-5R-3	GATACGTGACTGAAACTTAGGTTGTTG
Long UPM	CTAATACGACTCACTATAGGGCAAGCAGTGGTATCAACGCAGAGT
Short UPM	CTAATACGACTCACTATAGGGC
NUP	AAGCAGTGGTATCAACGCAGAGT
PU14-confirmF	AGTGAATTTCAGCAAAATTACCAGGAGT
PU14-confirmR	ATGGATACTCAGCAATGACGAATAGCGC

Primers for RT-PCR	
PU14-RT-F	AGGCGGGACAGCACAAAAACC
PU14-RT-R	GTTCATTTGGTTCATTTGGTTCA
Nacrein-F	GGCTTTGGCGACGAACCGGA
Nacrein-R	ACACGGGGGAGTGGTCAGGG
KRMP-F	AAGAAATGTCACCCTTGGGATTGG
KRMP-R	AATCATCGCCACCATATCCATCG
actin-F	GATGGTGCCGAGTATGTGGTA
actin-R	CGTTGATTATCTTGGCGAGTG

Primers for RNAi	
dsPU14-F	GCGTAATACGACTCACTATAGGGAGAGTAGCTCCAAATCAACAGAACAA
dsPU14-R	GCGTAATACGACTCACTATAGGGAGACTAAAGGTGATTGACCTCCAGC
dsGFP-F	GCGTAATACGACTCACTATAGGGAGATGTTCACCGGGGTGGTGCCCATCCT
dsGFP-R	GCGTAATACGACTCACTATAGGGAGATCGAACTTCACCTCGGCGCGGGTCT

Primers for pMAL-c5X plasmid construction	
c5X-PU14-F	GGATTTCACATATGTCCATGCAGTTTTCGGCCAAACAAGT
c5X-PU14-R	TAATTACCTGCAGGGAATTCTCAGTGGTGGTGGTGGTGGTGACGAAGAGGGAACGG
rPU14-F	GCCGCCAGCGGTCGTCAGACTGT
rPU14R	TCCGCTCCCGGCGGATTTGTCC

#### Confirmation of the Full-Length cDNA

The full-length cDNA sequence was confirmed using primers PU14-confirmF and PU14-confirmR. Bioinformatics software was then used to analyze the gene. The following websites were used: ORF Finder (http://www.ncbi.nlm.nih.gov/gorf/orfig.cgi), protein signal peptide prediction (http://www.cbs. dtu.dk/services/SignalP/), theoretical mass and theoretical pI prediction (http://web.expasy.org/compute_pi/), and protein secondary structure prediction (http://smart.embl-heidelberg.de/index2.cgi). Tandem-arranged repeat units were identified using the XSTREAM website (https://amnewmanlab.stanford.edu/xstream/).

### Expression and Distribution Pattern of *PU14*

cDNA templates from seven tissues were applied to detect the mRNA abundance of *PU14* by RT-PCR. cDNA templates were obtained by reverse-transcription of 500 ng total RNA in the following tissue, including foot, gonad, gill, mantle pallium, viscus, mantle edge, and adductor muscle, from four individuals using PrimeScript™ RT Master Mix (Perfect Real Time) (Takara, Japan). All PCR products were cloned and verified by sequencing.

Real-time quantitative PCR (qPCR) was used to quantify the expression level of *PU14*. The expression and distribution pattern of *PU14* in *P*. *fucata* was determined using SYBR Premix Ex Taq (Takara, Japan), following the manufacturer’s instructions in a LightCycler 480 system (Roche Diagnostics, Switzerland).

All the qPCR experiments used β-actin as the reference gene. qPCR was performed on a Roche LightCycler 480 PCR machine with PrimeScript™ RT Master Mix (Takara) using the primer pair RT-PU14-F/RT-PU14-R to amplify *PU14* gene fragments (see Table 1 for primer details). β-actin, the internal control, was amplified using Actin-F/Actin-R. KRMP (Liang et al. 2016) and nacrein (Miyashita et al. 2012), as positive controls, were respectively amplified using the primer pairs KRMP-F/KRMP-R and Nacrein-F/Nacrein-R. Cycle threshold (Ct) values were calculated in each reaction and normalized to the reference control, and relative gene expression was calculated using the comparative Ct method.

### Functions of *PU14* in Shell Restoration: Shell Notching Assay

Shell notching assays were performed following the protocol from Huang et al. (2007), with some modifications. We set eight groups of pearl oysters with five individuals each. Then, we cut the shell margin with a V-shape notch near the adductor muscle without touching the mantle tissue. At 0, 12, 24, 36, 48, and 72 h after notching, mantle tissues were immediately put into liquid nitrogen and stored after being cut and total RNA from all groups were extracted at 72 h. Mantle tissues from five oysters without any treatment were used as controls.

Real-time PCR was conducted to detect the expression of *PU14*. β-actin was used as a reference gene and matrix protein KRMP and nacrein were positive controls. All experiments were repeated three times.

### Gene Silencing by RNA Interference

#### dsRNA Synthesis

RNAi is an effective method to study a specific gene’s function as it can control the expression level of a single gene. In this study, we use an RNAi assay to study the function of *PU14* in the bio-mineralization process by silencing the expression of *PU14*. Specific primers dsPU14-F and dsPU14-R were designed according to the *PU14* coding region. Vector pEGF-N1 (NEB, USA) was used as a template to amplify GFP with primers dsGFP-F and dsGFP-R. PCR products were purified by EasyPure Gel Purification Kit (Transgene, China). Double-strand RNAs were synthesized using a RiboMAX Large Scale RNA Production System T7 kit (Promega, USA).

Three groups of five adult individuals each were set, in which the experimental group was injected with 60 μg PU14-dsRNA, while the other two groups were control groups injected with 60 μg GFP-dsRNA or Milli-Q water.

#### Gene Expression Analysis

A week later, we extracted RNA from the mantle tissue for gene expression analysis using qPCR following the same protocol as mentioned above. The shells of each individual were cleaned and air dried for microstructure observation.

#### Microstructure Observation of the Shells by Scanning Electron Microscopy

After gene silencing, we observed the inner surface structure of the shells. After cleaning and drying, the shells were cut into 0.5 cm × 0.5 cm pieces and coated with gold for 60 s before observation. All shells were examined and observed using a scanning electron microscope (SEM; FEI Quanta, 15 kV). At least 20 images were taken from each shell, and representative images were chosen.

### In Vitro Protein Expression and Purification

#### Plasmid Construction

We amplified the gene sequence of *PU14* without the signal peptide by PCR with primers c5X-PU14-F/c5X-PU14-R and then inserted the gene into the prokaryotic expression vector pMAL-c5X. We added a His_6_-tag in primer c5X-PU14-R. Then PU14 protein with a C-His tag was inserted downstream from the malE gene of *E*. *coli*., which encodes maltose-binding protein (MBP), in the pMAL-c5X vector (New England BioLabs Inc., NEB, USA), generating the recombinant vector pMAL-c5X-rPU14, which produced the corresponding fusion protein rPU14 with MBP at the C terminus of PU14. This fusion protein was more soluble in water and was expressed and purified for the CaCO_3_ in vitro crystallization assay.

#### Protein Expression and Purification

Purified plasmid pMAL-c5X-rPU14 was used to transform *E. coli* strain Transsetta (DE3) (Transgene, China) for expression. Transformed *E. coli* were cultured in LB medium at 37 °C and 200 rpm, induced with 0.6 mM isopropyl-β-D-thiogalactopyranoside (IPTG) when the OD_600_ reached 0.7–0.8, and then cultured at 37 °C and 200 rpm for another 12 h.

*E. coli* were collected by centrifugation at 6000* g* for 5 min at 4 °C and then suspended in lysis buffer (50 mM Tris, 100 mM NaCl, 5% glycerol, 1 mM DTT, 1 mM EDTA; pH 8.0). Next, to break the cell walls, cells were disrupted on ice using an ultrasonic dismembrator (Sonics & Materials Inc., USA) at 28% power with a 4s on and 6s off pulse cycle. After centrifugation at 12,000* g* for 40 min at 4 °C, the supernatant was removed and the insoluble fraction was used for purification. The insoluble fraction was resuspended and washed in denaturing lysis buffer (20 mM Tris, 500 mM NaCl, 6 M urea; pH 7.5).

Washed inclusion cell bodies were dispersed and dissolved in lysis buffer with 6 M urea (in binding buffer), and filtered supernatant was applied to a 1 mL Ni-NTA resin (Sangon Biotech, China) column. The column was washed with 30 mM imidazole in binding buffer. Then soluble, active rPU14 was eluted using elution buffer (20 mM Tris, 500 mM NaCl, 300 mM imidazole; pH 7.5), desalted to storage buffer (20 mM Tris, 500 mM NaCl; pH 7.5) using a Hitrap desalting column (GE Healthcare, USA), and stored at 4 °C until further analyses. Fractions were collected, boiled, and resolved by SDS-PAGE to confirm purification.

### In Vitro CaCO_3_ Crystallization Assay

There are two different carbonate crystals, calcite crystals and aragonite crystals. According to previous studies (Su et al. 2013; Towler 2003), we set two crystallization systems respectively. For the calcite crystallization system, the saturated calcium solution was prepared by mixing 100 mM NaHCO_3_ and 50 mM CaCl_2_ to a final Ca^2+^ concentration of 8 mM in Milli-Q water immediately before mixing with proteins. For aragonite crystallization system, Mg^2+^ was added to a final concentration of 50 mM.

The original concentration of MBP and rPU14 was 200 μg/ml, which was diluted into 4 and 40 μg/ml respectively. We obtained a total of 20 μl of saturated solution by mixing 5 μl protein samples with concentrations uniformly modified to 4, 40, and 200 μg/ml and 15 μl saturated calcium solution. MBP, water, and protein storage buffer were used as control.

Each sample was dripped onto the glass bottom of confocal dishes (Nest, China) and then incubated at room temperature for 24 h. After incubation, the calcite/aragonite was washed gently with Milli-Q water and protein storage buffer and then dried in the air. An FEI Quanta 200 SEM was used to image the crystals. The crystal form was characterized by the Raman spectrum and XRD. Raman spectroscopy was performed with an extinction wavelength at 514 nm and scanning range from 100 to 1500 cm.

Statistical Analyses. All figures were created using SigmaPlot 11.0 (Systat Software Inc., Germany) and Photoshop CC 2017 (Adobe, USA). The significant differences were calculated using Student’s *t* test. The statistical significance for all tests was set at *P* < 0.05. (*P < 0.05, **P < 0.01).

## Results

### Identification and Bioinformatic Analyses of *PU14* Gene

**Fig. 1:**
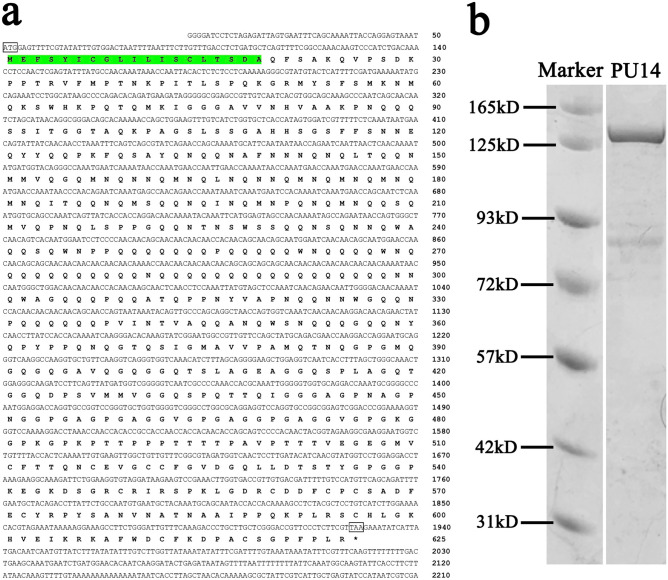
The cDNA sequence information of *PU14*. **a** Nucleotide sequence of PU14 of *P*. *fucata* obtained by RACE. **b** SDS- PAGE of recombinant protein PU14 with MBP tag at 120 kD

Applying RACE to clone the 5′ and 3′ flanking sequence, we obtained full-length *PU14* (GenBank accession number MT212404). The full-length *PU14* cDNA sequence is 2210 bp. It contains a 90-bp 5′-untranslated sequence, a 282-bp 3′-untranslated sequence, an in-frame ATG start codon, an in-frame TAA stop codon, and a 1878-bp ORF encoding a deduced 674-amino acid protein (Fig. 1a). A BLASTx search of the GenBank database revealed that *PU14* did not show significant similarity to any known genes from any other species.

Characteristics of PU14 protein were also predicted and analyzed. The deduced mature protein had a calculated molecular mass of 78 kDa, and the theoretical isoelectric point was 9.00. The amino acid composition of PU14 is Gln-rich, with 23.2% Gln, but poor in Asp and Glu content, which are rich in many other shell matrix proteins (Fig. S1c). SignalP 4.1 showed that *PU14* has a 19-amino acid signal peptide at the 5′ terminal, which is an important feature of shell matrix proteins (Fig. S1a). And it contains seven tandem repeats (Fig. S1c), which is also a feature of matrix proteins (Fang et al. 2011). Protein secondary structure prediction did not identify any obvious functional domains in *PU14*.

### Tissue-Specific Expression of *PU14*

**Fig. 2:**
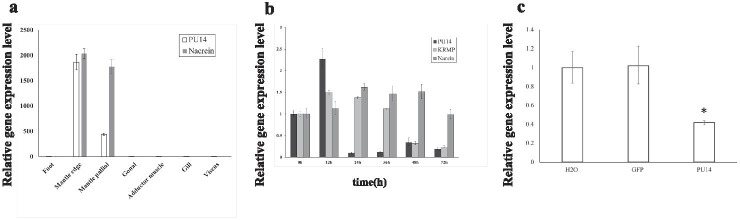
In vivo functions of *PU14*. **a** Gene expression pattern of PU14 in various tissues was determined by RT-PCR. **b** PU14 expression level after shell notching. Expression of negative control (0 h) is used as a relative value of 1.0. **c** PU14 expression inhibited by RNAi

In order to understand and evaluate potential functions of PU14 protein, *PU14* tissue-specific expression was measured by RT-PCR in seven tissues: foot, gonad, gill, mantle pallial, mantle edge, adductor muscle, and viscus. The results showed relatively higher *PU14* expression in mantle pallial and mantle edge. The expression in mantle pallial and mantle edge was several thousand-fold and several 100-fold higher, respectively, than that in the other tissues (Fig. 2a). Because mantle tissue plays a major role in shell formation, *PU14* expression there would be consistent with a related function.

### Function of *PU14* During Shell Formation In Vivo: Shell Notching

*P. fucata* shell will activate self-repair after the shell has been damaged, which is a stress response involving shell mineralization genes. To investigate *PU14* function during shell formation, we conducted notching experiments. *PU14* expression levels at 0 h, 12 h, 24 h, 36 h, 48 h, and 72 h after notching are shown in Fig. 2b. We also detected the expression levels of two other matrix protein genes, *Nacrein* and *lysine-rich matrix protein* (*KRMP*), as positive controls.

As shown in Fig. 2b and compared with the initial expression level at 0 h, *PU14* expression increased significantly to a maximum value at 12 h after notching and then decreased at later time points, indicating it may play a role in shell regeneration and formation shortly after shell damage.

### Function of *PU14* During Shell Formation In Vivo: RNAi Experiment

*PU14* function during shell formation was further analyzed using RNAi experiments. RNAi is an effective method for studying the function of a specific gene because it controls that gene’s expression level as a single variant. In this study, we knocked down *PU14* expression by injecting specifically designed double-stranded RNAs (dsRNA) into the adductor muscles of oysters. As controls, we also injected GFP dsRNA and water into two additional groups of oysters. Seven days after injection, total RNA was extracted from mantle tissues, and RT-PCR was performed to measure *PU14* expression.

Compared with the group injected with water, the expression level of *PU14* in the GFP dsRNA-injected group was similar. In contrast, *PU14* expression decreased by approximately 40% in the group injected with 60 μg PU14 dsRNA (Fig. 2c).

**Fig. 3:**
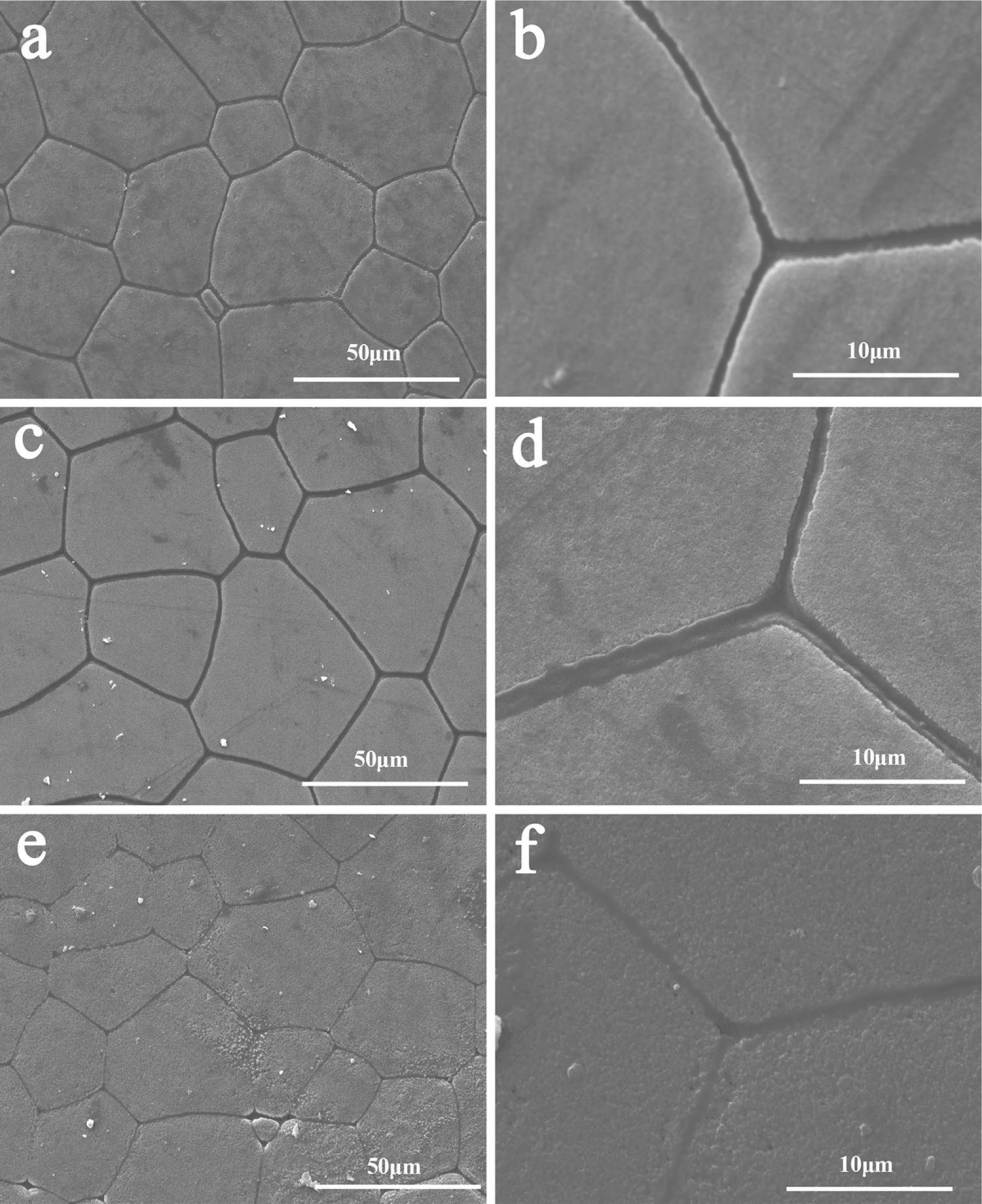
Effects of the inhibition of *PU14* on regulating the prismatic layer of inner shell. **a, b** ddH_2_O group: SEM images of the normal shell prismatic layers. **c**, **d** GFP dsRNA injected group: SEM images of the prismatic layers. **e, f** PU14 dsRNA injected group: SEM images of the prismatic layers

Shell surfaces were observed by SEM. Usually, after down-regulation of important matrix proteins, shell microstructures will be disrupted. In the GFP dsRNA injection control group, the prismatic layer showed normal prism structures with smooth surfaces and smooth and tight cracks between different prism tablets (Fig. 3a, b). The nacreous layer also showed a normal pattern, with nacreous tablets that were regular hexagonal structures. However, injections of 60 μg PU14 dsRNA caused the prismatic layer surface to be rougher (Fig. 3e–h), suggesting dual roles for PU14 during shell reparation and formation. The nacreous layer was not as severely disturbed as the prismatic layer (Fig. S2).

### Expression and Purification of Recombinant PU14 Protein

Several methods have been applied to express PU14 protein in *E. coli*. In order to obtain soluble protein, the recombinant vector pMAL-C5x was designed and successfully transformed into *E.coli* (Transsetta DE3). Large amounts of soluble protein were expressed. MBP-tagged PU14 (the recombinant protein PU14-MBP with a His_6_ tag on its C-terminal, rPU14) was obtained. According to the online tool (http://www.peptidesynthetics.co.uk/tools/), the predicted molecular mass of rPU14 was about 117 kDa, and the protein purification band was consistent with that prediction (Fig. 1b).

### Function of rPU14 During In Vitro Calcium Carbonate Crystallization

The in vitro calcium carbonate crystallization assay has been widely used to mimic biomineralization during shell formation. We used a reaction system containing saturated Ca(HCO)_2_ solution, constituted by mixing Ca^2+^ and HCO^−^ solution to an ultimate Ca^2+^ concentration of about 8 mM, and then added rPU14 protein to the system to explore its effects on CaCO_3_ crystal morphology and polymorphism.

**Fig. 4:**
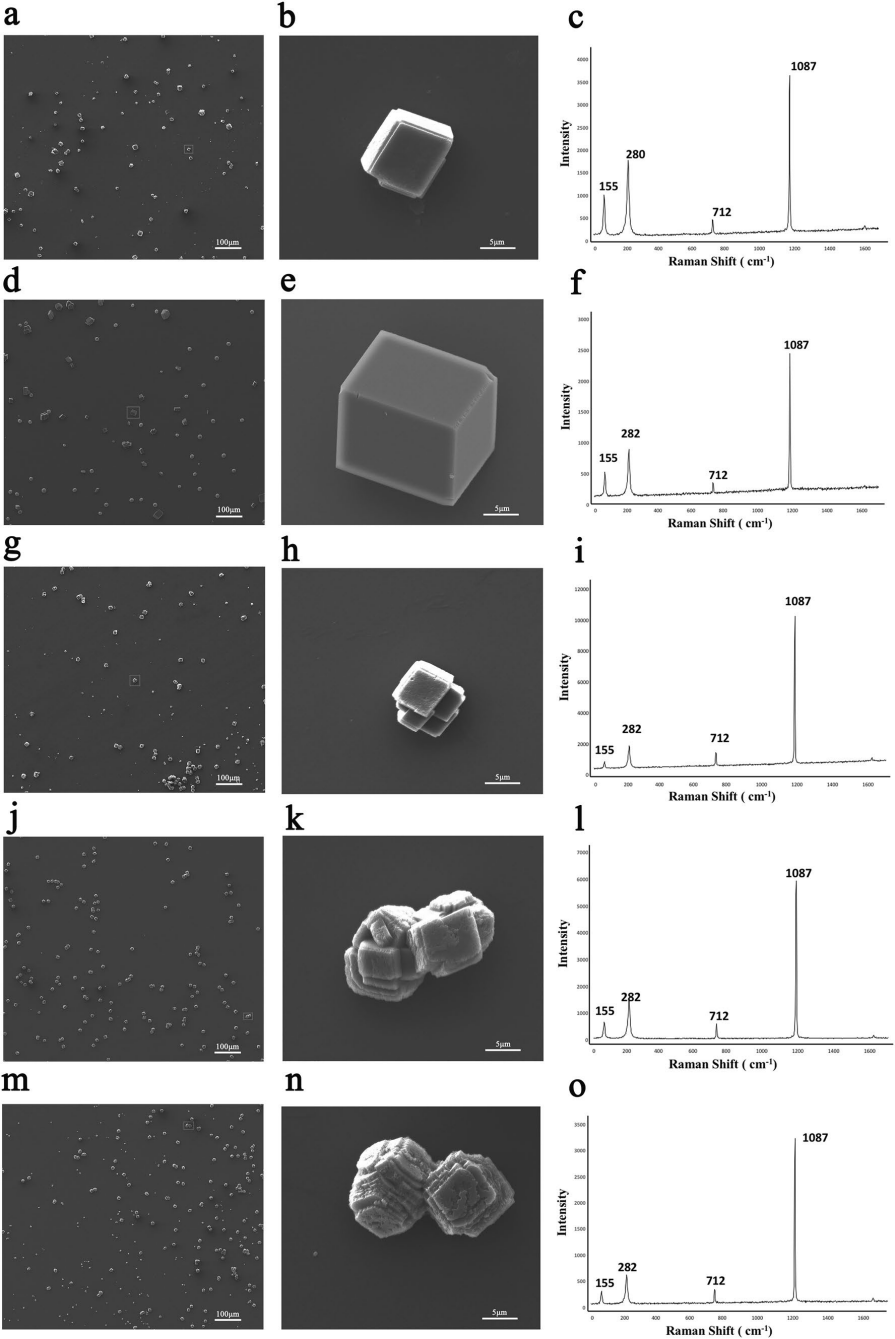
SEM images and Raman spectra of in vitro calcite crystallization in the presence of rPU14. **a, b** Crystals grown in the presence of Tris NaCl buffer; **d, e** 80 μg/ml MBP; **g, h** 4 μg/ml rPU14; **j, k** 40 μg/ml rPU14; **m, n** 200 μg/ml rPU14. **b, e, h, k,** and **n** The amplifications of the crystals indicated by red boxes in **a, d, g, j,** and **m**), respectively. **c, f, i, l,** and **o** The Raman spectrum of the crystals, respectively

Compared with the typical rhombohedral calcite crystals seen with the control group under buffer (Fig. 4a and b), the crystals made in the presence of 200 mg/mL MBP showed no obvious morphological alterations (Fig. 4d and e). However, crystals in the PU14 groups showed significantly altered crystal morphologies, with the degree of alteration increasing with increasing rPU14 concentrations. At a low rPU14 concentration (1 μg/20μL; Fig. 4g and h), the crystals were slightly changed compared with the control group. Several rhombohedral crystals appeared within one crystal in place of typical calcite crystals with clear edges. At increased rPU14 concentration, the rhombohedral faces of the crystals were rougher and extra growth structures arose irregularly on all crystal faces (Fig. 4j, k, m, and n).

Raman spectra indicated that all crystals were calcite forms with characteristic peaks at around 282, 712, and 1087 cm^−1^ (Fig. 4c and f). These data suggest that rPU14 may play critical roles in regulating calcite morphology but do not cause phase transformations in the calcite polymorph.

Additionally, magnesium was introduced into the crystallization system to induce aragonite formation, the main component of the nacreous layer. Standard aragonite carbonates are ball- or spindle-shaped and are comprised of many needle-like spindles (Fig. S2a and b). The addition of rPU14 did not change the morphology or crystal form (Fig. S2d-l). Raman spectra showed that the crystals in rPU14, buffer, and MBP groups were all aragonites and no phase transformation was detected.

These data show that rPU14 may play critical roles in regulating crystal morphology, especially calcite crystal formation, which is the main component of Pearl oyster prismatic layers.

## Discussion

As previous studies have shown, matrix proteins play important roles in the biogenesis of various biominerals, including pearl oyster *P. fucata* shell formation. Several matrix proteins, including Pif (Suzuki et al. 2009; Du et al. 2016), Nacrein (Miyashita et al. 2012), KRMP (Liang et al. 2015), and PfN23 (Fang et al. 2012), have been investigated and demonstrated to regulate shell biomineralization via various mechanisms.

In a previous report, PU14 was identified as a candidate matrix protein by liquid chromatography-tandem mass spectrometry (LC-MS/MS) analysis of proteins extracted from *P. fucata* shells (Liu et al. 2015a, b). It existed in prismatic layer and was insoluble in EDTA. Subsequently, microarray analysis of global gene expression during *P. fucata* larval development showed that PU14 expression increased from the umbonal stage to the juvenile stage, which is the main period of shell formation (Liu et al. 2015a, b).

We conducted several experiments and analyses, identifying PU14 as a matrix protein and proving its function during shell formation. First, we cloned the *PU14* gene using RACE and deduced its protein. PU14 protein has a 19-amino acid signal peptide at its N-terminal, indicating secretory post-translation processing (Fig. S1a). Sequence analysis indicated that PU14 is a Gln-rich protein (Fig. S1c), which is similar to the previously certified matrix protein PfY2 (Yan et al. 2017). Several kinds of amino acids (i.e., Glu, Asp, Gln, Asn, Lys, Thr, and Tyr) are considered to be crucial for regulating polymorphism, size, and aggregation of CaCO_3_ depositions (Samata et al. 1999; Yan et al. 2017). Repeated domains are a common feature of matrix proteins, and the content, composition, or arrangement of these seem to influence protein functions (Liu et al. 2015a, b).

Usually, matrix proteins are secreted by the mantle tissue, close to the inner side of the shell. Studies have shown that mantle edge and mantle pallial in mantle tissues are connected to prismatic layer formation and nacreous layer mineralization, respectively (Kono et al. 2000; Checa et al. 2009; Fang et al. 2011; Heinemann et al. 2011; Liao et al. 2019). Tissue-specific *PU14* expression analysis showed that its expression level is hundreds of times higher in mantle pallial than in foot and gonad tissues (Fig. 2a), which is consistent with the fact that PU14 protein was mainly localized to the prismatic layer.

However, it is also highly expressed in mantle edge, indicating a possible nacreous layer function. Until now, only a few *P. fucata* matrix proteins, including KRMP-3 (Liang et al. 2015), MSI7 (Feng et al. 2009), Alv (Kong et al. 2018), and PfY2 (Yan et al. 2017), have been shown to have dual roles in aragonite and calcite formation in vitro. Most matrix proteins function in one layer, either the prismatic layer or the nacreous layer. Because shell formation is a complex process with multiple genes participating and interacting, *PU14* may associate with other shell matrix genes during nacreous layer formation. It should be noticed that recent studies of *PfN23* (Fang et al. 2012) and *PfN44* (Pan et al. 2014) have shown that specific protein secretion regions do not always directly correlate with function. The correlation between protein localization and shell layer formation functions needs to be studied further.

PU14 was detected in an EDTA insoluble fraction of the prismatic layer. According to previous studies, proteins in the EDTA-insoluble fraction are thought to localize around calcium carbonate crystals and participate in shell framework formation (Heinemann et al. 2006). Yet, in recent years, researchers have found that these proteins are also involved in biomineralization regulation (Liang et al. 2016). We assume that PU14 could be soluble initially and play function in prismatic layer. However, it becomes insoluble after being secreted outside to form the shell.

We assessed *PU14* function in *P. fucata* adults in vivo using shell notching and RNAi analyses. Notching damages shells, inducing a response that mimics natural shell formation processes, including up-regulation of biomineralization-related genes. After notching treatment, *PU14* mRNA levels rapidly rose until 12 h, and then decreased to lower levels (Fig. 2b). However, these expression changes were not completely synchronized with *Nacrein* and *KRMP* expression changes in the mantle pallial and edge, respectively. It is reasonable to conclude that increases in promotive matrix proteins accelerate shell formation. For example, Nacrein protein that accumulates after notching might elevate the carbonic anhydrase activity that produces hydrogen carbonate needed for crystal formation. The *PU14* expression profile indicates a potential function during early stages of shell formation. RNAi is also an effective way to detect functions of matrix proteins in vivo (Fang et al. 2012; Liang et al. 2015; Pan et al. 2014; Suzuki et al. 2009). RNAi interference of *PU14* showed that decreased *PU14* expression resulted in abnormal prismatic layer surfaces (Fig. 3). Shell formation is a complex process, and we speculate that PU14 protein might cooperate temporally and spatially with other proteins to regulate well-organized biomineralization.

In order to explore the effects of PU14 protein on CaCO_3_ crystallization in vitro, experiments were also conducted with purified recombinant protein rPU14. After adding rPU14 to calcite crystallization solutions, the morphology of calcite particles changed visibly and a dosage effect was observed with increasing protein concentration. In contrast, aragonite aggregation was not obviously changed. We also measured Raman spectra of these particles, and the results showed that rPU14 did not change the crystal form (Fig. 4). Thus, PU14 may be considered as a CaCO_3_ morphology regulatory factor. It has been reported that matrix proteins can be distributed around CaCO_3_ surfaces or be integrated inside of crystals during in vivo biomineralization (Fu et al. 2016). Further study should be done to explore protein preferences for specific crystal faces and its influence on crystallization rates. We speculate that PU14 interacts with immobilized ions to influence CaCO_3_ formation.

In conclusion, PU14, a matrix protein that exists in *P. fucata* prism and nacre, plays important roles in shell formation. In this study, we cloned a full-length *PU14* cDNA and explored *PU14* function in vivo. We also observed PU14 regulation of calcite crystal morphology in vitro. The observations indicate a function in the prismatic layer of *P. fucata* shells, providing insights into prismatic layer formation. Further, these results lay a foundation for future studies of this protein’s molecular mechanism, which would provide a new perspective on biomineralization.

## Supplementary Information

Below is the link to the electronic supplementary material.

Fig. S1. PU14 protein characteristics (**a**) signal peptide predication of PU14 protein by signalP 4.0 (**b**) PU14 Tandem repeats, the colorful boxes indicate repeats. (**c**) PU14 amino acid composition (TIFF 7.08 MB)

Figure S2. SEM images and Raman spectra of in vitro aragonite crystallization in the presence of rPU14. (**a**, **b**) Crystals grown in the presence of Tris NaCl buffer; (**d**, **e**) 80 μg/ml MBP; (**g**, **h**) 4 μg/ml rPU14; (**j**, **k**) 40 μg/ml rPU14. (**b**, **e** ,**h** ,**k**) were the amplifications of the crystals indicated by red boxes in (**a**, **d**, **g**, **j**), respectively. (**c**, **f**, **i**, **l**) showed the Raman spectrum of the crystals, respectively. (TIF 2.0 MB)
