# Supplementary figures and images for: PU14, a Novel Matrix Protein, Participates in Pearl Oyster, Pinctada Fucata, Shell Formation
Source: Mar Biotechnol (NY). 2021 Mar 10;23(2):189–200. doi: 10.1007/s10126-020-10014-3 (PMC8032588; doi:10.1007/s10126-020-10014-3)

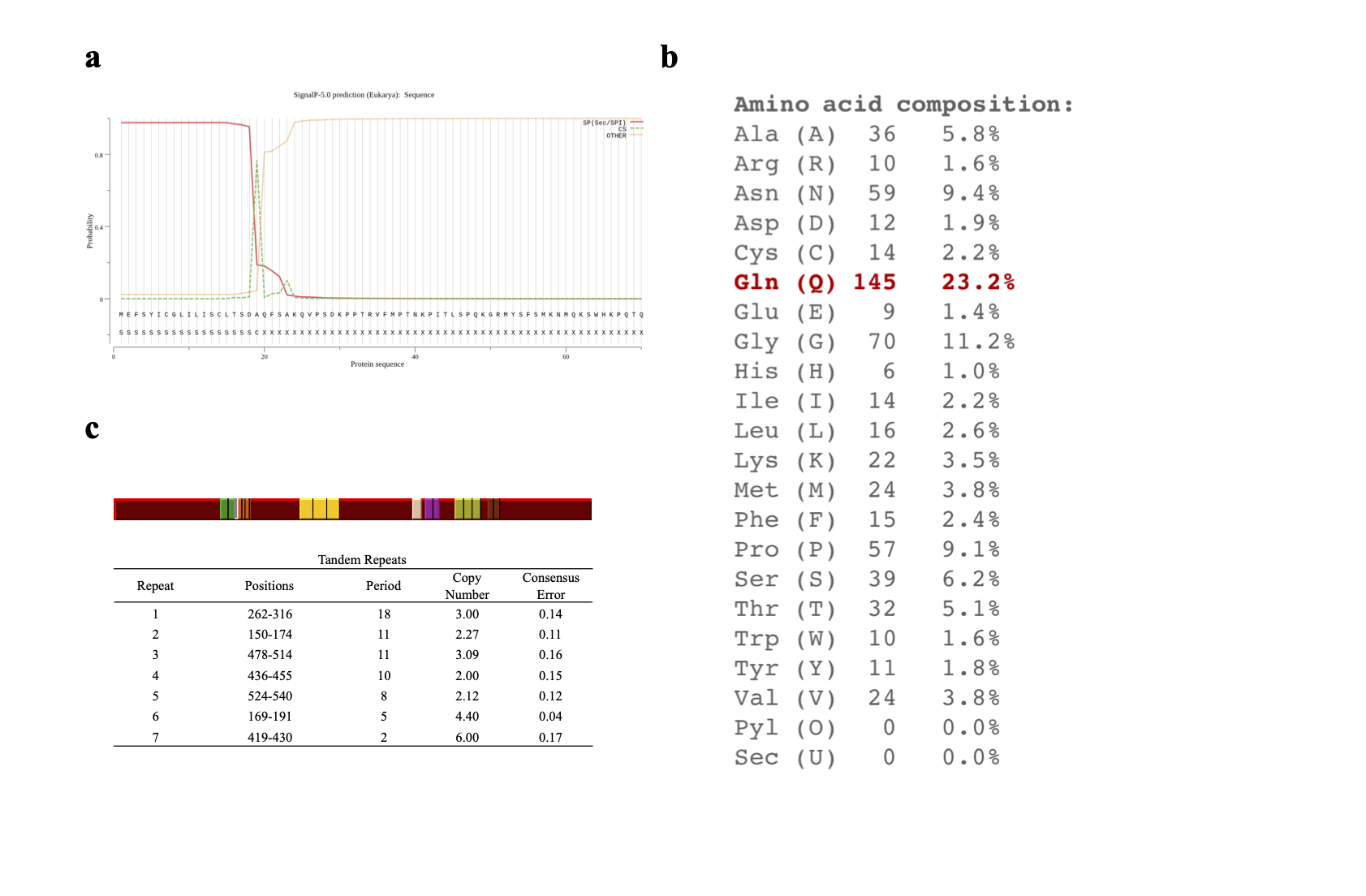

Supplement: Supplementary file 1 — Fig. S1. PU14 protein characteristics (a) signal peptide predication of PU14 protein by signalP 4.0 (b) PU14 Tandem repeats, the colorful boxes indicate repeats. (c) PU14 amino acid composition (TIFF 7.08 MB) [file 10126_2020_10014_MOESM1_ESM.tiff]

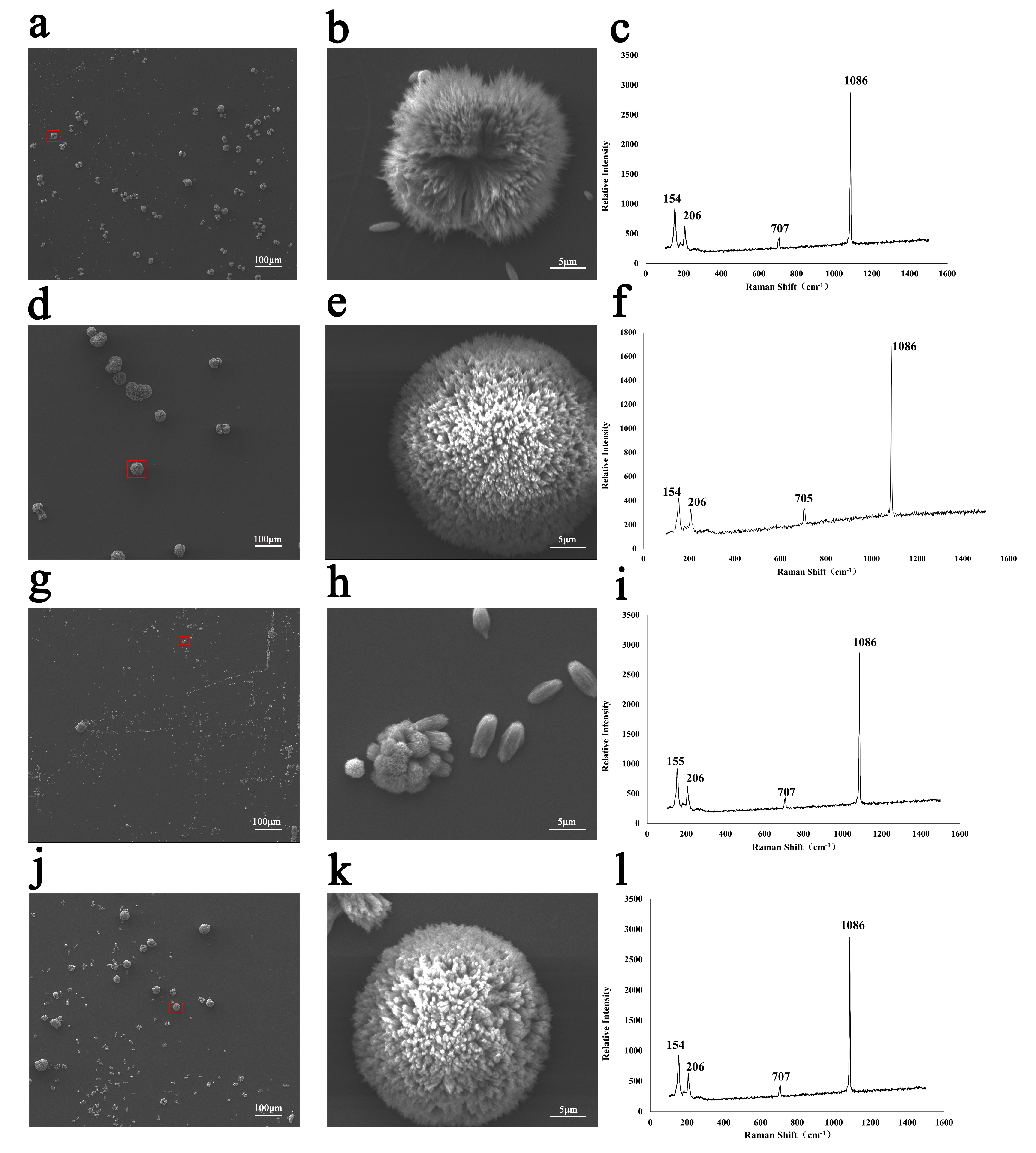

Supplement: Supplementary file 2 — Figure S2. SEM images and Raman spectra of in vitro aragonite crystallization in the presence of rPU14. (a, b) Crystals grown in the presence of Tris NaCl buffer; (d, e) 80 μg/ml MBP; (g, h) 4 μg/ml rPU14; (j, k) 40 μg/ml rPU14. (b, e ,h ,k) were the amplifications of the crystals indicated by red boxes in (a, d, g, j), respectively. (c, f, i, l) showed the Raman spectrum of the crystals, respectively. (TIF 2.0 MB) [file 10126_2020_10014_MOESM2_ESM.tif]
